# Editorial: Mechanisms for the Alteration in the Crosstalk Among Insulin-Sensitive Tissues

**DOI:** 10.3389/fendo.2022.883659

**Published:** 2022-04-14

**Authors:** Michele Schiavon, Amalia Gastaldelli

**Affiliations:** ^1^ Department of Information Engineering, University of Padova, Padova, Italy; ^2^ Institute of Clinical Physiology, Italian National Research Council, Pisa, Italy

**Keywords:** insulin resistance, metabolomics (OMICS), lipidomics, glucotoxicity, lipotoxicity, liver, skeletal muscle, adipose tissue

Insulin plays a key role in energy homeostasis by exerting anabolic functions in different target (insulin-sensitive) tissues such as skeletal muscle, liver, and adipose tissue (1). Insulin resistance is defined as a shift of the concentration-effect curve towards higher insulin levels, but it is usually also combined with a reduction of the maximal insulin responsiveness (2).

In addition to the well-known mechanisms resulting from an increased caloric intake and decreased physical activity (3), insulin resistance has also recently been shown to be driven by alterations in the crosstalk between different tissues (4, 5). This results from multiple metabolites, which not only serve as substrates in metabolism but also as mediators in metabolic pathways regulating insulin resistance and finally contributing to the onset and evolution of many metabolic diseases encompassing the spectrum of the metabolic syndrome, like obesity, cardiovascular disease (CVD), nonalcoholic fatty liver disease (NAFLD), type 2 diabetes (T2D), etc. (6). Hence, investigating the role of the key players like glucose and its derivatives, lipids, amino acids, and other metabolites, both in normal as well as pathologic state, is of primary importance for better characterizing the etiology of many different diseases as well as the development of new and effective therapeutic agents.

Here follows the aim of this Research Topic, where the authors contributed with original and review articles aiming to investigate the mechanisms of alteration in metabolic-mediated crosstalk among insulin-sensitive tissues. This was achieved by means of *in vitro* and/or *in vivo* animal/human studies and different methodological techniques, including gene expression, metabolomics, and lipidomics, as well as standard surrogate measurements used in large clinical studies (7).

Starting from the original work by Cao et al. the authors investigated the impact of exogenous ATP/ADP/AMP administration on glucose regulation in a mice model, both from a whole-body as well as cellular point of view, by means of metabolomics. In fact, while the excess of intracellular ATP was supposed as a possible risk factor for insulin resistance, little was known about the role of extracellular ATP and its derivatives. With this study, the authors concluded that elevated extracellular ADP levels induce, through direct and indirect mechanisms, the promotion of hepatic gluconeogenesis, and this was also intensified in the context of insulin resistance. In particular, they also found that increased hepatic gluconeogenesis was coupled with elevated NADH levels, possibly representing a marker for the pathogenesis of insulin resistance in the liver.

Then, an original work by Qin et al. investigated the role of insulin resistance in the onset of nephrolithiasis and its recurrence. By means of multivariate logistic regressions, the authors found a positive association between the triglyceride-glucose index, a surrogate index positively correlated with insulin resistance (8), and the risk of the onset and recurrence of kidney stones. The authors speculated that this could be driven by increased levels of free fatty acids and/or insulin per se, usually occurring in the context of insulin resistance.

Three works focused on skeletal muscle mass and function and its relationship with lipotoxicity. Armandi et al. reviewed the mechanisms leading to sarcopenia, i.e. loss of skeletal muscle mass (muscle atrophy), quality, and strength, often associated with aging but recently identified to also be associated with metabolic diseases like obesity, T2D, NAFLD, and strictly related to insulin resistance. In particular, the review offers an overview on the alterations of metabolic pathways affecting the skeletal muscle due to abnormal functioning of adipose tissue, liver, and gut, thus highlighting the strict association between sarcopenia and metabolic diseases, with glucotoxicity and lipotoxicity as main drivers.

The original work by Pasmans et al. investigated the crosstalk between liver and muscle and the association between NAFLD and sarcopenia by studying the effect of high-fat diet and hepatic steatosis on muscle mass and function. They identified several hepatokines secreted by fatty livers possibly implicated in the development of sarcopenia that, once incubated with skeletal muscle cells (C2C12 myoblasts) contributed to muscle insulin resistance and to the alteration of muscle genes implicated in anatomical structure and function. Nevertheless, as reported by the authors, future studies are also needed to assess the opposite mechanism, i.e. if secretion products from an unhealthy muscle can affect liver health.

The other original study by Tran et al. investigated the role of 1-deoxysphingoliplids (1-DSLs), an atypical class of sphingolipids found significantly elevated in the plasma of individuals with impaired fasting glucose, metabolic syndrome (MetS), and T2D and recently indicated as early predictors of T2D (9). By means of *in vitro* experiments, the authors showed that 1-DSLs directly compromise the functionality of C2C12 myoblasts and their action is more potent than saturated fatty acids as palmitate by inducing cytotoxic effects both on skeletal muscle precursors, as well as differentiated cells, and a significant reduction in insulin-stimulated glucose uptake. However, as reported by the authors, further studies are needed to confirm these results and, if the case, modulation of 1-DSL levels could be investigated as a complement to the available therapies in T2D.

Another interesting review from Laurila et al. reports the pleiotropic effects of secretin, a re-discovered hormone that should be studied as a target for the treatment of obesity. The authors highlight the role of secretin in the crosstalk between the gut, where the hormone is predominantly secreted by the S-cells in the duodenum, and other organs: in the white adipose tissue it stimulates lipolysis, in the brown adipose tissue it activates thermogenic effect thus increasing energy expenditure, in the brain, due to its action on attenuating the anticipatory reward responses to appetizing food, it increases satiation and delays resumption to eat.

Finally, in Ahlin et al. the authors investigated the mechanisms and mediators involved in the metabolic improvements achieved after metabolic surgeries (Roux-en-Y gastric bypass and biliopancreatic diversion) other than weight loss. In particular, by using an integrative approach combining adipose tissue gene expression with plasma metabolite profiling (metabolomics), the authors identified significant changes in twelve metabolites and adipose tissue mRNA levels after metabolic surgery that were also associated with changes in lipid, insulin, and glucose levels. Of interest, among the metabolites, they found significant changes in 2-hydroxybutyric acid, valine, glutamic acid, that were previously found associated with insulin resistance and fatty liver disease (10, 11). As reported by the authors, if confirmed in other study cohorts, the metabolites and metabolic pathways found in this work may be used in the future as biomarkers for assessing metabolic improvements after metabolic surgery.

We thank all the authors contributing to this Research Topic for their great work in reviewing as well as helping the advancement of this very complex but fascinating world of metabolic crosstalk in humans.

## Author Contributions

The editorial article has been written, edited, and reviewed by MS and AG. All the authors listed in the Editorial have made a substantial, direct, and intellectual contribution to the work. All authors contributed to the article and approved the submitted version.

## Funding

MS was supported by MIUR (Italina Minister for Education) under the initiative “Departments of Excellence” (Law 232/2016). AG received the financial support from the European Union’s Horizon 2020 Research and Innovation Programme under the Marie Skłodowska-Curie Grant Agreement No. 734719 (mtFOIE GRAS project), and from the Italian Ministry of Research for the project PRIN-2017 Protocol No.  2017KAM2R5_004.

## Conflict of Interest

The authors declare that the research was conducted in the absence of any commercial or financial relationships that could be construed as a potential conflict of interest.

## Publisher’s Note

All claims expressed in this article are solely those of the authors and do not necessarily represent those of their affiliated organizations, or those of the publisher, the editors and the reviewers. Any product that may be evaluated in this article, or claim that may be made by its manufacturer, is not guaranteed or endorsed by the publisher.
